# Rasagiline-induced severe recurrent hypoglycemia in a young woman without diabetes: a case report

**DOI:** 10.1186/s13256-017-1202-x

**Published:** 2017-02-02

**Authors:** Fawzi A. Bachet Ibrahim, Fauzia Rashid, Azza A. Bin Hussain, Fatheya Alawadi, A. Bashier

**Affiliations:** Endocrine Division, Dubai Hospital, Dubai, United Arab Emirates

**Keywords:** Rasagiline, Parkinson disease, MAO inhibitor, Hypoglycemia, Diabetes, Case report, SSRI

## Abstract

**Background:**

We report a case of a patient with recurrent severe hypoglycemia after initiating the drug rasagiline (Azilect) for Parkinson disease.

**Case presentation:**

A 25-year-old Emirati woman who had been diagnosed with Parkinson disease due to a genetic mutation since the age of 18 years presented to our hospital. She had been treated with a rotigotine patch 2 mg per day along with carbidopa + levodopa + entacapone 25 mg/100 mg/200 mg (Stalevo) over these years. Recently, her Stalevo had been changed to rasagiline (a monoamine oxidase B inhibitor). Soon after this change, she started experiencing recurrent documented severe hypoglycemia requiring hospitalization. Her hypoglycemic symptoms completely disappeared after 5–7 days of drug withdrawal. Despite detailed evaluation, no other causal relationship was documented except for rasagiline.

**Conclusions:**

To the best of our knowledge, this case report documents an unknown association between rasagiline and hypoglycemia.

## Background

Severe hypoglycemia is a frequently encountered medical emergency, seen commonly in patients with diabetes who are taking drugs such as insulin or insulin secretagogues (e.g., sulfonylurea) [1]. Management of these cases usually involves parenteral glucose infusion, hypoglycemic dose adjustment, and treatment of underlying precipitating medical conditions.

Other than iatrogenic hypoglycemia in patients with diabetes, hypoglycemia can be reactive hypoglycemia or secondary to endocrine causes such as adrenal insufficiency, pituitary insufficiency, and insulinoma. Hypoglycemia can also be seen with severe systemic diseases such as sepsis, renal failure, and hepatic failure. It has also been reported with many medications, including many non-antidiabetic drugs [2].

Drugs can affect glucose homeostasis because of their peculiar pharmacokinetics or pharmacodynamic drug interactions, causing an alteration in secretion or action of different endogenous chemicals such as insulin, glucagon, catecholamines, growth hormone, and cortisol [3]. Many drugs have been reported as a cause of hypoglycemia, including quinine, beta blockers, and angiotensin-converting enzyme (ACE) inhibitors, as well as antidepressants such as selective serotonin reuptake inhibitors (SSRIs) and monoamine oxidase inhibitors (MAOIs) [4].

Considering the magnitude of this problem, it is imperative to understand the underlying pathophysiological mechanisms by which a non-antidiabetic drug can cause hypoglycemia. This allows for more predictable, practical, and safe health care practices and avoids unnecessary investigations as well. We report a case of a patient with recurrent hypoglycemic events following the introduction of the MAOI rasagiline. These attacks improved dramatically after withdrawal of the medicine. To the best of our knowledge, this is the first reported case of hypoglycemia in association with rasagiline.

## Case presentation

We present a case of a 25-year-old Emirati woman who has been diagnosed with Parkinson disease (PD) related to homozygous parkin mutation since the age of 18 years. She was being treated with a rotigotine patch 2 mg per day along with carbidopa + levodopa + entacapone 25 mg/100 mg/200 mg (Stalevo; Novartis Pharmaceuticals, East Hanover, NJ, USA) twice daily. She has no other comorbid condition. Owing to the patient’s concerns about future side effects of Stalevo, she was shifted to rasagiline 1 mg in September 2015. Three days later, after initiation of the drug, she experienced a fainting attack and collapsed while at work. Her capillary blood sugar in the ambulance was low (48 mg/dl), so she was given intravenous 50% dextrose, which resolved her symptoms completely. She was kept in observation for 24 h and discharged to home before complete evaluation owing to the patient’s social issues.

She continued to have frequent similar episodes of dizziness, tremors, and sweating at home and work for the next 3 weeks, which were relieved by sugar intake. These symptoms were not associated with a drop in blood pressure, palpitations, or flushing. There was no significant change in her weight or bowel habits, nor did she report any changes in meal type or frequency. Episodes of hypoglycemia occurred in both fed and fasting states. The lowest documented reading was 48 mg/dl. She had never experienced any hypoglycemic event before during her life. She was not taking any other medications apart from those mentioned above.

Clinically, she was fully alert and oriented to time, place, and person. Her cranial nerve examination, including the field of vision, was unremarkable. She had minimal resting tremors, more on the right side than the left. Her postural reflexes were intact. She had few dyskinetic movements of her neck and hand. She also experienced torticollis with a head turn to the right. She also has intermittent eye-closing spasms and excessive blinking with mild blepharoclonus and possible mild levator inhibition. She had some dystonic cramping of her feet. She had normal bulk, tone, power, and reflexes in all four limbs with bilateral downgoing plantar response. The results of her sensory and cerebellar examination were unremarkable, her gait was normal, and she had no postural drop of blood pressure and no pigmentation to suggest adrenal insufficiency. The results of the rest of the systemic examination were essentially normal.

Owing to the factors described above, it was strongly suspected that rasagiline might be a cause of the patient’s symptoms, because MAOIs previously had been documented to cause hypoglycemia. Rasagiline was stopped, and an evaluation was done to rule out any other underlying cause, such as insulinoma or Addison’s disease. We performed a 72-h fasting hypoglycemia test, a short Synacthen test, and urinalysis for sulfonylurea screening.

Laboratory investigations revealed the following: white blood cell count 5.3 × 10^3^/μl, hemoglobin 14.2 g/dl, platelets 244 × 10^3^/μl, urea 9 mg/dl, creatinine 0.5 mg/dl, sodium 140 mmol/L, potassium 3.9 mmol/L, calcium 8.7 mg/dl, albumin 4.1 g/dl, alanine transaminase 20 U/L, alkaline phosphatase 70 U/L, HbA1C 4.6%, free thyroxine 12.5 pmol/L (normal range 11.5–22.7 pmol/L), free triiodothyronine 4.5 pmol/L (normal range 3.5–6.5 pmol/L), thyroid-stimulating hormone 1.92 μIU/ml (normal range 0.55–4.78 μIU/ml), antiendomysial antibody (immunoglobulin G [IgG]) <1 U/ml (normal range <7 U/ml), endomysium antibody (IgA) <1 U/ml (normal range <7 U/ml), tissue transglutaminase IgA <2.0 RU/ml (negative <20 RU/ml), tissue transglutaminase IgG 0.03 RU/ml (normal range negative <1.0 RU/ml), insulin autoantibodies negative <0.4 U/ml (normal range <0.4 U/ml), oral antidiabetic drugs in urine (screening) negative; Synacthen test normal, 72-h fasting test normal response with blood glucose of 45 mg, serum insulin 3.7 IU/ml (normal range 4.0–16.0 IU/ml), serum proinsulin <0.1 pmol/l (normal range <11), C-peptide 0.8 ng/ml (normal range 1.8–4.7 ng/ml). Imaging studies, including magnetic resonance imaging of the pancreas and ultrasound of the abdomen and pelvis, were normal.

The patient was observed closely during her stay, and she gradually stopped experiencing hypoglycemia over a period of 1 week, with a minimum sugar reading of 78 mg/dl. After stopping of rasagiline, our patient felt much improvement, with home sugar readings between 70 and 110 mg/dl and no further symptomatic hypoglycemia.

## Discussion

The best way to confirm a drug’s relation to any particular side effect is by withdrawal and rechallenging with the medicine to prove recurrence of the symptoms. In our patient, we could not perform withdrawal/rechallenge to further strengthen this causal relationship between rasagiline and hypoglycemia owing to the associated severe health risk. However, sudden-onset hypoglycemic episodes after introducing rasagiline to a patient with otherwise isolated PD without any documented autonomic neuropathy, in addition to her complete recovery from these hypoglycemic episodes after withdrawal of the drug, strongly suggest rasagiline as a culprit agent behind her symptoms.

Rasagiline mesylate is a novel second-generation propargylamine developed for the management of PD [5]. Data have confirmed the drug’s selectivity for monoamine oxidase B (MAO-B) and that the drug is not metabolized to amphetamine and methamphetamine, which are toxic by-products of the metabolism of selegiline, another selective MAO-B inhibitor, and cause of many untoward cardiovascular and neurological side effects [6, 7]. Rasagiline has been proved to be a safe drug with infrequent cardiovascular or psychiatric side effects [8, 9].

Drug-induced hypoglycemia secondary to non-antidiabetic agents is a well-identified side effect. Some of the following absorption facts were observed in a systematic review published in 2009 by the Hypoglycemia Task Force of the Endocrine Society after review of all related literature about drug-induced hypoglycemia. It involved 50 (11%) randomized controlled trials and 25 (6%) case-controlled or controlled cohort studies, as well as case reports or single-cohort studies (83%). In this review, the authors reported that none of the 164 drugs associated with hypoglycemia were supported by high-quality evidence. The most commonly responsible drugs were from (1) the quinolone group, as cited in 32 publications comprising an aggregate of 826 patients; followed by (2) quinine, which is a well-known culprit with hypoglycemia, documented by 30 publications comprising an aggregate of 326 patients; then by (3) beta blockers, cited in 49 publications comprising an aggregate of 131 patients; and then by (4) ACE inhibitors, reported by 11 publications comprising an aggregate of 129 patients [4].

Antidepressant drugs such as SSRIs and MAOIs are also reported to be associated with hypoglycemia, especially in patients with diabetes who are taking insulin [10]. MAOs are the first class of antidepressant, discovered in 1952. They work by irreversibly blocking MAO, which is an enzyme ubiquitously present in the body. MAO comes in two forms: MAO-A and MAO-B.

MAO-A works by oxidative deamination of dopamine, serotonin, and norepinephrine, whereas MAO-B metabolizes phenylethylamine and dopamine. The inhibition of these enzymes by MAOs leads to increased concentrations of serotonin, dopamine, and norepinephrine and is responsible for the MAOIs’ antidepressant effects [11]. There are some compounds in this group that nonselectively inhibit MAO enzymes, such as tranylcypromine and phenelzine, as well as some selective blockers of MAO-B, such as selegiline and rasagiline. Some data in the past had shown that nonselective MAOs may potentiate hypoglycemia, especially in patients with diabetes [12]. There is also a case report in the literature about selegiline-induced hypoglycemia in a patient with PD in whom it took 1 week to recover from hypoglycemia [13]. Although the half-life of selegiline is 1.5 h after single-dose administration, it is impossible to predict the extent of MAO-B inhibition from steady-state plasma levels. It has been reported that the parent compound, as well as the metabolite, accumulates after multiple doses, and the metabolite also has an MAO-inhibitory property, so prolonged action may also be expected owing to the irreversible mechanism of action [14].

Despite the fact that hypoglycemia has been documented to occur with many antidepressants, including nonselective MAOIs, there is no documented report of altered regulation of glucose metabolism by rasagiline [7]. All of the hydrazine-type MAOIs potentiate glucose-stimulated insulin secretion. Of the nonhydrazine inhibitors, only harmine and α-methyltryptamine potentiated glucose-stimulated insulin secretion [15].

During our literature search, we found many reports of hypoglycemia seen after use of antidepressant drugs, including SSRIs, which have been found to interfere with blood glucose metabolism, increasing the risk of hypoglycemic episodes, especially in patients with diabetes [16, 17]. In one retrospective analysis, the effect of antidepressants was noticed in control of diabetes, and it was concluded that, in animal models, hypoglycemia resulting from phenelzine, a hydrazine MAOI, is due to direct inhibition of gluconeogenesis secondary to hydrazine structure homology, not due to its function as an MAOI. In these models, serotonergic effects in the presence of MAOI potentiated hypoglycemia, causing as much as a 30% decrease in fasting plasma glucose, whereas dopamine and norepinephrine influences in these models appear to be hyperglycemic [18].

In a review, Goodnick *et al*. [19] recommended the use of SSRIs in patients with diabetes and depression because they enhance insulin sensitivity and glucose use compared with tricyclic antidepressants. In another case report, sertraline, which is an SSRI, was given to a young woman with for premenstrual dysphoric disorder and no previous history of glucose intolerance. This resulted in recurrent episodes of hypoglycemia, which resolved after discontinuation of the drug. Sertraline has been shown to blunt postprandial hyperglycemia in studies done in rats, and it also potentiates the hypoglycemic effect of sulfonylurea in patients with diabetes [20]. We could not find any genetic association between PD and hypoglycemia, nor can we explain the possible cause of hypoglycemia in our patient, because she was not taking any concurrent medicine, including SSRIs, which could have been a cause of increased insulin sensitivity, as discussed above. However, it can be speculated that, being a member of the MAOI group, albeit quite selective, rasagiline may cause hypoglycemia owing to the same pathophysiological mechanism previously described for MAOIs (i.e., rasagiline): that it causes increased serotonergic effects that result in hypoglycemia by enhancing insulin sensitivity, insulin release, and decreased gluconeogenesis.

## Conclusions

To the best of our knowledge, rasagiline has not previously been known as a cause of hypoglycemia in patients with no previous glucose dysregulation. We believe that our present case report is the first to describe such a relationship. The cause-and-effect relationship between the two needs to be evaluated further.

## Abbreviations

ACE: Angiotensin-converting enzyme
Ig: Immunoglobulin
MAO: Monoamine oxidase
MAOI: Monoamine oxidase inhibitor
SSRI: Selective serotonin reuptake inhibitor
TCA: Tricyclic antidepressant
